# Supplementary material for: Content-rich biological network constructed by mining PubMed abstracts
Source: BMC Bioinformatics. 2004 Oct 8;5:147. doi: 10.1186/1471-2105-5-147 (PMC528731; doi:10.1186/1471-2105-5-147)
Supplement: Additional File 2 — The original results of the above study (non-essential files are deleted to keep the file size under the limit set by BMC bioinformatics). [file 1471-2105-5-147-S2.bz2 › chilibotAdditionalFile2/dip20/361ID8360477E5749/html/IL6ST_IL11.html]

 


 **IL6ST** and **IL11** 
  
Found 219 abstracts in PubMed, retrieved 20.  
 

 What does Google say? 
 PDF only 
| .edu only 

---

**Interactive relationship** (e.g. stimulation, inhibition, etc)

**Inhibitory relationship**- Here we show that MDL A has a highly selective inhibitory effect on IL 6 and IL 11  [ **IL11** ]  activities by inhibiting a gp130  [ **IL6ST** ]  activity while suppressing bone loss in ovariectomized mice.  Ref: 12417753 Proc Natl Acad Sci U S A, 2002
- Osteoclastogenesis induced by IL 6 and IL 11  [ **IL11** ]  was inhibited by the addition of an anti gp130  [ **IL6ST** ]  antibody but not by osteoprotegerin.  Ref: 12584029 Bone, 2003
- Here we show that Cdk9 interacts with gp130  [ **IL6ST** ] , the receptor of the Interleukin 6 IL 6 family of cytokines, which includes Leukemia Inhibitory Factor LIF, Oncostatin M OSM, Ciliary Neurotrophic Factor CNTF, Interleukin 11  [ **IL11** ]  IL 11  [ **IL11** ]  and Cardiotrophin CT 1.  Ref: 12386808 Oncogene, 2002
**Neutral relationship**- IL 11  [ **IL11** ]  belongs to the gp130  [ **IL6ST** ]  cytokine family, which includes also IL 6.  Ref: 12697042 J Endocrinol, 2003
- IL 11  [ **IL11** ]  forms a high affinity complex, thereby inducing gp130  [ **IL6ST** ]  dependent signaling.  Ref: 12865319 Endocrinology, 2003
- IL 11  [ **IL11** ]  is a member of the gp130  [ **IL6ST** ]  family of cytokines, which signal via assembly of multisubunit receptor complexes containing at least one molecule of the transmembrane signaling receptor gp130  [ **IL6ST** ] .  Ref: 12865319 Endocrinology, 2003

**Non-interactive relationship** (e.g. studied together, co-existance, homology, etc.)

- OBJECTIVE The development of megakaryocytes is regulated by thrombopoietin TPO, which binds to the c mpl receptor, and by several other cytokines such as interleukin IL 6, IL 11  [ **IL11** ] , leukemia inhibitory factor LIF, cilliary neurotropic factor CNTF, and oncostatin OSM, which bind to gp130  [ **IL6ST** ]  protein coupled receptors.  Ref: 12135673 Exp Hematol, 2002
- Leukemia inhibitory factor LIF, oncostatin M OSM, interleukin IL 6, and IL 11  [ **IL11** ]  regulate hemopoiesis and LIF, OSM, and IL 6 also inhibit the proliferation of myeloid leukemic cell lines through the signal transducing subunit gp130  [ **IL6ST** ] .  Ref: 12543079 Cytokine, 2002
- Interleukin IL 6, leukaemia inhibitory factor LIF and IL 11  [ **IL11** ]  belong to the same family of cytokines whose receptors utilize gp130  [ **IL6ST** ]  as the signalling molecule.  Ref: 12200462 Mol Hum Reprod, 2002
- ELISA was used to determine the serum levels of soluble interleukin 2 receptors, interleukin 1  [ **IL11** ] , interleukin 6, and 2 soluble interleukin 6 receptors sIL 6R and soluble gp130  [ **IL6ST** ]  in 18 patients with cluster headache 6 women and 12 men during the cluster period and in 17 healthy controls who were headache free 3 women and 14 men.  Ref: 12864761 Headache, 2003
- In liquid cultures, megakaryocytes from gp130  [ **IL6ST** ]  delta delta mice are smaller than those from wild type mice and do not increase in size upon stimulation with interleukin 6 or interleukin 11  [ **IL11** ] .  Ref: 12423677 Exp Hematol, 2002
- These and others like IL 11  [ **IL11** ]  and ciliary neurotrophic factor are referred to as the gp130  [ **IL6ST** ]  cytokines because they share the gp130  [ **IL6ST** ]  glycoprotein as a common receptor initial signal transducer.  Ref: 12538632 Endocrinology, 2003
- OnM induced desensitization also prevents responses to other gp130  [ **IL6ST** ]  signaling cytokines e.g. leukemia inhibitory factor and interleukin 11  [ **IL11** ] .  Ref: 12724316 J Biol Chem< MedlineTA>J Biol Chem, 2003
- Recombinant human Interleukin 11  [ **IL11** ]  rhIL 11, Neumega TM, a gp130  [ **IL6ST** ]  signalling cytokine approved for treatment of thrombocytopenia, also raises von Willebrand factor VWF and factor VIII FVIII by an unknown mechanism.  Ref: 12649145 Blood< MedlineTA>Blood, 2003
- Administration of either interleukin 6 or interleukin 11  [ **IL11** ]  to gp130  [ **IL6ST** ]  delta delta mice failed to increase platelet numbers, despite an increase in the production of megakaryocytes.  Ref: 12423677 Exp Hematol, 2002
- To our surprise, none of the other gp130  [ **IL6ST** ]  protein related cytokines tested IL 11  [ **IL11** ] , LIF, CNTF, and OSM activated these signaling pathways in CD34 cells, megakaryoblasts, or platelets.  Ref: 12135673 Exp Hematol, 2002
- Previous studies have identified three distinct receptor binding sites, I, II, and III, crucial for the binding of murine IL 11  [ **IL11** ]  mIL 11 to both the IL 11R and gp130  [ **IL6ST** ] .  Ref: 12865319 Endocrinology, 2003
